# THE CULTURE OF FAMILY CAREGIVING OF OLDER ADULTS AMONG AFRICAN AND AFRO-CARIBBEAN IMMIGRANTS TO THE US AND CANADA

**DOI:** 10.1093/geroni/igae098.0456

**Published:** 2024-12-31

**Authors:** Cleopatra Kum, Casey Phillips, Lynn Warner, Islam Banisalman, Maria Roche-Dean

**Affiliations:** University of Cincinnati, Cincinnati, Ohio, United States; University of Kansas Medical Center, Kansas City, Kansas, United States; University of Cincinnati Libraries, Cincinnati, Ohio, United States; University of Cincinnati, Cincinnati, Ohio, United States; University of Kansas, Kansas City, Kansas, United States

## Abstract

An increasing number of African and Afro-Caribbean immigrants to the United States and Canada are aging. Older adults are dealing with various health conditions, which makes them increasingly dependent on family members and friends for their health and social needs. The assistance they receive includes help with physical care, transportation, medication assistance, and emotional and social support, amongst others. Caregivers play a pivotal role in maintaining the health of older adults of African and Afro-Caribbean immigrants in the United States and Canada. However, very little is understood about the expectations and culture of family caregiving within this group. This study explores the culture of family caregiving of older adults amongst African and Afro-Caribbean immigrants in the United States and Canada. We conducted a scoping review of the literature with the assistance of two experienced health science librarians using CINAHL, PubMed, Embase, and OVID MEDLINE. Preliminary findings show that while some articles discussed aspects of family caregiving on the African continent, none were specific to family caregivers of older adults living in the United States and Canada. While it is important to understand the cultural implications of family caregiving within African communities, there are nuances of the African and Caribbean diaspora that have serious implications for the health of older adults. This study adds to our understanding of family caregiving among African and Afro-Caribbean immigrants. It points to a need for tailored intervention for supporting family caregivers within African and Afro-Caribbean immigrant communities.

